# A protocol for periprosthetic joint infections from the Northern Infection Network for Joint Arthroplasty (NINJA) in the Netherlands

**DOI:** 10.1186/s42836-022-00116-9

**Published:** 2022-04-11

**Authors:** W. P. Zijlstra, J. J. W. Ploegmakers, G. A. Kampinga, M. L. Toren-Wielema, H. B. Ettema, B. A. S. Knobben, P. C. Jutte, M. Wouthuyzen-Bakker, the Northern Infection Network for Joint Arthroplasty (NINJA), A. Al Moujahid, P. F. Doorn, B. L. E. F. ten Have, G. Mithoe, L. E. Pirii, I. N. Vlasveld, M. Stevens, M. G. A. van Vonderen, A. J. de Vries

**Affiliations:** 1 Department of Orthopedic Surgery, Medical Center Leeuwarden, Leeuwarden, the Netherlands; 2 Department of Orthopedic Surgery, University of Groningen, University Medical Center Groningen, Groningen, the Netherlands; 3 Department of Medical Microbiology and Infection Prevention, University of Groningen, University Medical Center Groningen, Groningen, the Netherlands; 4 Department of Pharmacy, University of Groningen, University Medical Center Groningen, Groningen, the Netherlands; 5 Department of Orthopedic Surgery, Isala Clinics, Zwolle, the Netherlands; 6 Department of Orthopedic Surgery, Martini Hospital, Groningen, the Netherlands

**Keywords:** Periprosthetic joint infection (PJI), Arthroplasty Surgery, Treatment, Protocol, Debridement Antibiotics and Implant Retention (DAIR)

## Abstract

Periprosthetic joint infection (PJI) is a devastating complication of joint arthroplasty surgery. Treatment success depends on accurate diagnostics, adequate surgical experience and interdisciplinary consultation between orthopedic surgeons, plastic surgeons, infectious disease specialists and medical microbiologists. For this purpose, we initiated the Northern Infection Network for Joint Arthroplasty (NINJA) in the Netherlands in 2014. The establishment of a mutual diagnostic and treatment protocol for PJI in our region has enabled mutual understanding, has supported agreement on how to treat specific patients, and has led to clarity for smaller hospitals in our region for when to refer patients without jeopardizing important initial treatment locally. Furthermore, a mutual PJI patient database has enabled the improvement of our protocol, based on medicine-based evidence from our scientific data. In this paper we describe our NINJA protocol.

**Level of evidence:** III

## Background

Periprosthetic joint infections (PJI) are a devastating complication of joint arthroplasty surgery. Treatment success depends on accurate diagnostics, adequate surgical experience and interdisciplinary consultation among orthopedic surgeons, plastic surgeons, infectious disease specialists and medical microbiologists. In order to facilitate this interdisciplinary collaboration and enhance treatment success in our region in the North of the Netherlands, we initiated the Northern Infection Network for Joint Arthroplasty (NINJA). The NINJA network started in 2014 and consisted of a few dedicated orthopedic surgeons aiming to enhance the treatment of PJI in our region, including three large teaching hospitals (Medical Center Leeuwarden, Martini Hospital Groningen, Isala Clinics Zwolle) and one academic hospital (University Medical Center Groningen). Over the years, collaboration with infectious disease specialists and medical microbiologists intensified, and a mutual diagnostic and treatment protocol was established. It was agreed that all four hospitals adopted these strategies into their clinical practice. In addition, patients treated in all four hospitals were followed-up and evaluated to determine the treatment outcome. The protocol is named the NINJA protocol, and it has been further adopted by the smaller hospitals in our region as well, and has been recently updated.

The aim of this paper is to describe the NINJA protocol, and to share our common experience regarding the diagnosis and treatment of PJIs over the last years.

### Clinical aspects

An infection of a joint prosthesis can be broadly classified into the following three categories [1]:

1. **Early, Post-Surgical Infection:**

* ≤ 3 months after the placement of the joint prosthesis.
* Usually caused by intraoperative or postoperative colonization of bacteria through the wound.
* May present acutely with fever, significant wound leakage and a persistent high C-reactive protein (CRP). This acute presentation is usually caused by virulent microorganisms (particularly *S. aureus* and Gram-negative rods). The infection may also present chronically with a low CRP and minimal but persistent wound leakage. This chronic presentation is usually caused by low virulent microorganisms (especially coagulase negative *Staphylococci* [*Staphylococci* other than *S. aureus*] and *Enterococci*).

2. **Late Acute Hematogenous Infection:**

* Can occur at any stage, but usually > 3 months after placement of the joint prosthesis.
* Hematogenous seeding caused by a source of infection elsewhere, *e.g*. from a cellulitis or urinary tract infection in which the joint prosthesis becomes secondarily infected.
* Characterized by acute pain and swelling of the joint, with or without the presence of fever, in a previously asymptomatic joint. Generally caused by virulent microorganisms (especially *S. aureus*, *Streptococci* and Gram-negative rods).

3. **Late Chronic Infection:**

* > 3 months after the placement of the joint prosthesis.
* Usually caused by intraoperative or postoperative colonization of bacteria through the wound.
* Characterized by persistent pain at the joint prosthesis with or without loosening of the prosthetic joint. Generally caused by low virulent microorganisms originating from the skin (especially coagulase negative *Staphylococci*, *Corynebacterium* species and/or *Cutibacterium acnes* (formerly known as *Propionibacterium acnes*).

The aforementioned distinction is important with regard to the diagnosis, the empirical antibiotic treatment and the surgical treatment plan.

### Diagnostics

#### Periprosthetic Joint Infection definition

Several diagnostic criteria have been established to diagnose a PJI [2–4]. A PJI can be diagnosed with a high sensitivity and specificity [5] on the basis of the diagnostic criteria introduced in 2018 during the International Consensus Meeting in Philadelphia.

The prosthesis is considered to be infected if at least one of the following major criteria is satisfied:

1. A sinus tract with evidence of communication with the joint or visualization of the prosthesis OR
2. A minimum of two positive cultures of the same microorganism (with the same resistance pattern)
  In addition, the prosthesis is also considered infected when at least 6 points are scored on the following minor criteria and should be considered as *possibly infected* when 4 to 5 points are scored:
3.
  a. Serum erythrocyte sedimentation rate (ESR) > 30 mm/h (1 point)
  b. Serum CRP > 10 mg/L OR serum D-Dimer > 860 ng/mL (2 points);
  c. Leukocytes in synovial fluid > 3000 × 10^6^/L OR leukocyte esterase > 2 plus (3 points);
  d. Neutrophil granulocytes in synovial fluid > 80% (2 points);
  e. Positive alpha-defensin in synovial fluid (3 points);
  f. Synovial CRP > 6.9 mg/L (1 point);
  g. A single positive culture (2 points);
  h. Neutrophil influx of the peri-prosthetic tissue on histopathological examination (> 5 neutrophils per field in 5 high-power fields at 400 × magnification) (3 points).
    i. Intraoperative purulence (3 points).

However, when applying the above diagnostic criteria, it is good to realize that low-grade infections can still be missed as indicated by the recently published diagnostic criteria of the European Bone and Joint Infection Society EBJIS [4]. For this reason, the clinical context should always be taken into account. In particular, when synovial fluid markers are positive and/or in the case of positive histology for infection but the cultures remain negative, the following should be taken into consideration:

(i) Are there alternative explanations for the positive minor criteria? (*e.g*., metallosis, gout, active rheumatoid arthritis).
(ii) Have maximum efforts been made to optimize culture yield? (*e*.*g*., the use of sonication, are enough biopsies taken, and is the incubation time of cultures long enough?).

If, after answering the above questions, the suspicion of an infection is still high, additional diagnostic efforts should be made to find the causative microorganism (*e*.*g*., by means of molecular and serological diagnostics). The subsequent antibiotic treatment of these infections should be decided in a multidisciplinary team.

It should be noted that the cut-off values of the minor criteria can only be used if it is determined more than 12 weeks after index surgery. The biomarker calprotectin [6] is not part of the above diagnostic criteria but can be included in the decision-making process (see diagnostic flow charts). For example, a low calprotectin in synovial fluid has a very high negative predictive value and can be used to exclude a chronic PJI prior to revision surgery [6].

### Preoperative diagnostic workup

**Fig. 1:**
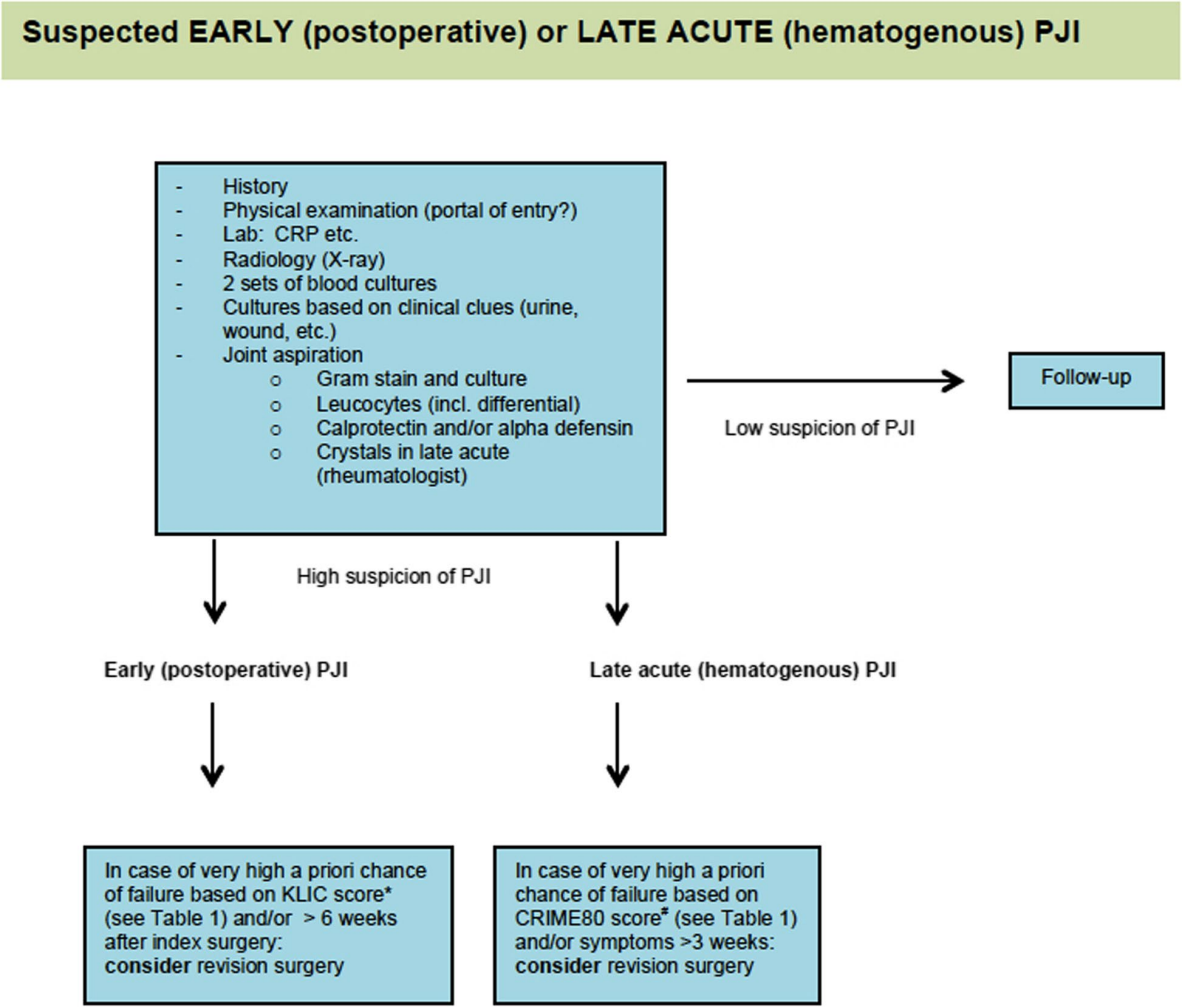
Flowchart of suspected early (postoperative) or late acute (hematogenous) PJI. *KLIC: Kidney, Liver cirrhosis, Index surgery, C-reactive protein and Cemented prosthesis (see Table 1) [7, 8]. ^#^CRIME80: Chronic obstructive pulmonary disease, Rheumatoid arthritis, Index surgery, Male gender, Exchange of mobile components and age > 80 years (see Table 1) [9]

**Fig. 2:**
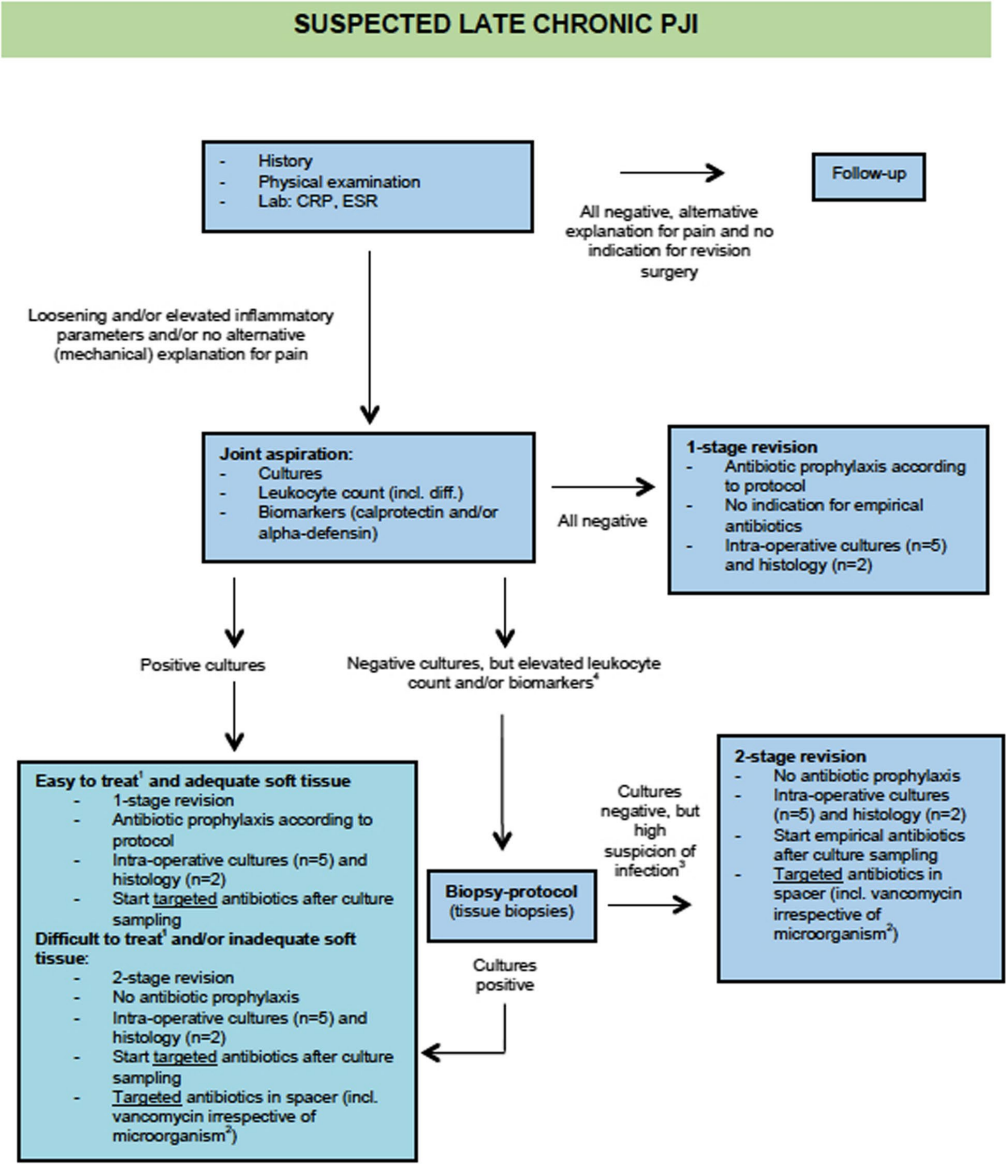
Flowchart of suspected late chronic PJI. ^1^Difficult to treat: chinolon resistant Gram-negative rods, rifampin-resistent *Staphylocci*, *Enterococci*, fungi and yeasts. ^2^In order to avoid secondary spacer infections with coagulase-negative *Staphylococci* [10]. ^3^For example in case of positive histology. ^4^Consider a nuclear bone or white blood cell scintigraphy if available. A bone scintigraphy is advised as a first step if the patient is > 5 years after the index surgery for knees and > 2 years for hips; when the affected prosthesis is younger, a white blood cell scintigraphy can be considered. If the bone scintigraphy is negative, infection is practically ruled out and no additional scans are needed. If the bone scan is positive, a white blood cell scintigraphy should be considered as it is more specific in diagnosing infection. If the white blood cell scintigraphy is negative, an infection is highly unlikely; if it is positive, cultures and histology should be performed as indicated above

The flowcharts below should be applied to patients with a suspected early (postoperative) or late acute (hematogenous) PJI (Fig. 1) and late chronic PJI (Fig. 2). For exact definitions, please refer to: 'clinical aspects'.

**Table 1:** Preoperative risk scores, predicting the failure of a DAIR procedure in the case of an unknown microorganism

KLIC-score^a^ (early PJI)			CRIME80-score^b^ (late acute PJI)		
**Variable**	**Description**	**Score**	**Variable**	**Description**	**Score**
**K**	Chronic renal failure (kidney)	2	**C**	COPD	2
				CRP > 150 mg/L	1
**L**	Liver cirrhosis	1.5	**R**	Rheumatoid arthritis	3
**I**	Index surgery (revision surgery or prosthesis indicated for a fracture)	1.5	**I**	Index surgery (prosthesis indicated for a fracture)	3
**C**	Cemented prosthesis	2	**M**	Male gender	1
	C-reactive protein > 115 mg/L	2.5			
			**E**	Exchange of mobile component	-1
			**80**	Age > 80 years	2
	**Risk of DAIR failure (%)** ^c^	Score^c^		**Risk of DAIR failure (%)** ^c^	**Score** ^c^
	28%	** ≤ 2**		22%	‑1
	37%	**2.5 – 3.5**		28%	0
	49%	**4 – 5**		40%	1 – 2
	55%	**5.5 – 6.5**		64%	3 – 4
	86%	** ≥ 7**		79%	≥ 5

^a^Based on a cohort of early acute PJI patients in Medical Center Leeuwarden, Martini Hospital and University Medical Center Groningen (*n* = 386)[8]

^b^Based on a cohort of late acute PJI patients in an international multicentre study (*n* = 340)[9]

^c^In case of *S. aureus*, the risk of failure with a score of -1 is already 44%[9]

### Preoperative diagnostic workup

Preferably, all cultures should be obtained without antibiotic treatment. If the patient is already being treated with antibiotics, the treatment should be discontinued for at least two weeks in patients with a chronic infection undergoing revision surgery, in order to obtain reliable culture results. Antibiotic prophylaxis should be administered prior to surgical incision, as this has hardly any influence on the culture yield and timely prophylaxis is important for the prevention of (secondary) infections [11].

In the case of an acute infection, tissue biopsies are taken intraoperatively. If the infection is chronic, the aim is to determine the causative agent preoperatively. Since the specificity of a positive culture of synovial fluid is high (~ 95%) [12], it can be stated with reasonable certainty that this is the causative microorganism of the PJI (unless there is a sinus tract with a high probability of a polymicrobial infection). In case of doubt and/or in the case of a negative culture of synovial fluid, preoperative tissue biopsies can be obtained in a sterile setting. In this scenario, the patient is given anesthesia and the orthopedic surgeon aspirates synovial fluid and obtains 5 peri-prosthetic tissue biopsies for culture and 2 peri-prosthetic tissue biopsies for histology. By this approach, the causative microorganism is isolated in approximately 10% of patients with a negative culture of synovial fluid [13].

When collecting intraoperative cultures, 5 tissue biopsies should be taken around the joint prosthesis, and synovial fluid should be aspirated for culture and biomarkers [14]. If available, all removed hardware should be sent for sonication [15] [incl. the head/polyethylene in the case of a DAIR (debridement and implant retention) procedure and spacer in the case of a 2-stage revision].

### Surgery

The indications for a DAIR, one-stage revision or two-stage revision are shown in Figs. 1 and 2. In general, acute infections are treated surgically with debridement, antibiotics and implant retention (DAIR). However, there are several factors that reduce the success of debridement while retaining the prosthesis in an acute infection. These include preoperative factors (see Table 1), the duration of symptoms, the type of microorganism and intraoperative factors (*e*.*g*., if the mobile components cannot be replaced, this will lead to a poorer outcome). If the a priori chance of success is very low according to the preoperative risk score (Table 1), a one- or two-stage revision may be considered. This should be discussed within the multidisciplinary team and with the patient.

Operative step-by-step plan for surgical debridement:

1. Time-out and set-up as primary prosthetic implant. Antibiotic prophylaxis should be administered prior to surgical incision to protect the prosthesis from (secondary) infections.
2. Open arthrotomy. Consider scar excision. Performing arthroscopy is contraindicated as this leads to inferior outcomes.
3. Take at least 5 deep tissue cultures at the site of infection (fluid, periprosthetic tissue, capsule, interphase tissue, bone if necessary). Clean instrumentation should be used for every biopsy. Subcutaneous cultures, wound swabs, or sinus tract cultures are contra-indicated.
4. Perform extensive debridement, with excision of all “suspicious” or necrotic tissues, including, if needed, synovectomy. Interchangeable prosthetic parts are removed in order to properly debride the joint (only if this does not present a risk of damage to the prosthesis) and sent for sonication if available in the treating hospital.
5. The remaining parts of the prosthesis are polished with a wet gauze or scrubbing sponge to macroscopically remove biofilm as much as possible. Use povidone-iodine (>2 min.), and at least 3 liters of saline with pulse lavage.
6. In principle, no gentamicin beads or gentamicin fleeces are left behind [16].
7. After debridement, gloves are changed, and with clean instruments, the new mobile prosthesis components are inserted (*e*.*g*. head and possibly polyethylene liner of the total hip prothesis, tibial insert of the total knee prosthesis). The wound is closed in layers. In general, no drains are left behind.

If the DAIR fails, a second DAIR can be performed in the case of persistent or recurrent wound leakage, redness of the wound suspected of infection, fever, and/or persistent elevated inflammatory markers without any alternative explanation, provided that the soft tissues are intact. In our cohort, the success rate after a second DAIR procedure for acute PJI was 75% and the prosthesis could be retained in more than 80% of patients [17]. After a failed second DAIR, it is advised to remove the prosthesis.

The surgical treatment for a chronic infection is a one- or two-stage revision surgery. A two-stage over a one-stage revision is preferred in case the causative microorganism is unknown in patients with a high suspicion of a PJI, in the case of difficult-to-treat microorganism or poor soft tissue condition (*e*.*g*., presence of a sinus tract, affected soft tissue due to radiotherapy, *etc*.). In the case of a two-stage revision, reimplantation takes place 6 weeks after prosthesis extraction under antibiotic therapy, unless otherwise decided in the multidisciplinary team.

### Antimicrobial therapy

#### Empirical and targeted antibiotic therapy

Table 2 (empirical antibiotic therapy in the case of an yet-unknown causative microorganism) and Table 3 (targeted antibiotic therapy in the case that the causative microorganism(s) is known) provide a guideline for the antibiotic regimen. The choice should always be made in consultation with the medical microbiologist and/or internist-infectiologist, based on the local epidemiology. The susceptibility of the microorganism should be known before initiating targeted treatment.

**Table 2:** Empirical antibiotic therapy (based on local epidemiology)

Type of infection	Antibiotic^b^	Dosage^a^
**Early (postoperative)**	Cefuroxime	1.500 mg IV *q.i.d*. or 1.500 mg loading dose, followed by 6.000 mg/24 h continuous infusion
	*plus*	
	Vancomycin	20 mg/kg loading dose, followed by 30 mg/kg/24 h continuous infusion (with adjustments based on blood level monitoring)
	*In case of sepsis add*	
	Tobramycin	7 mg/kg once daily (with adjustments based on blood level monitoring)
**Late acute (hematogenous)**	Cefuroxime	1.500 mg IV *q.i.d*. or 1.500 mg loading dose, followed by 6.000 mg/24 h continuous infusion
	*In case of sepsis add*	
	Tobramycin	7 mg/kg once daily (with adjustments based on blood level monitoring)
**Late chronic**	Ceftriaxone	2.000 mg once daily IV *or* 2.000 mg loading dose, followed by 2.000 mg/24 h continuous infusion
	*plus*	
	Vancomycin	20 mg/kg loading dose, followed by 30 mg/kg/24 h continuous infusion (with adjustments based on blood level monitoring)

^a^The above doses are based on adequate kidney function and a normal weight/BMI. In case of deviating values, contact the hospital pharmacist for dosing advice

^b^In the case of revision surgery also administer antibiotic prophylaxis in accordance to protocol; BMI ≤ 40 kg/m^2^: cefazolin 2 g, BMI > 40 kg/m^2^: cefazolin 3 g IV prior to surgical incision

**Table 3:** Targeted antimicrobial therapy based on microorganisms [20–26]

Microorganism	Antibiotic	Dosage^a^	Duration^c^
***S. aureus* or flucloxacillin-sensitive coagulase-negative** ***Staphylococci*** **(CoNS)**	Flucloxacillin	2.000 mg loading dose, followed by 12.000 mg/24 h continuous infusion IV	1–2 wks
	plus		
	Rifampin^b^	450 mg *b.i.d*. oral	1–2 weeks
	followed by:		
	Moxifloxacin or	400 mg *q.d*. oral	10–11 weeks
	Levofloxacin	500 mg *b.i.d*. oral	
	plus		
	Rifampin^b^	450 mg *b.i.d*. oral	10–11 weeks
**Methicillin resistent *S. aureus* or flucloxacillin coagulase-negative** ***Staphylococci* (CoNS)**	Vancomycin	20 mg/kg loading dose, followed by 30 mg/kg/24 h continuous infusion (with adjustments based on blood level monitoring)	1–2 weeks
	plus		
	Rifampin^b^	450 mg *b.i.d*. oral	1–2 weeks
	followed by:		
	Moxifloxacin *or*	400 mg *q.d*. oral	10–11 weeks
	Levofloxacin	500 mg *b.i.d*. oral	10–11 weeks
	plus		
	Rifampin^b^	450 mg *b.i.d*. oral	10–11 weeks
	In the case of chinolon resistance:		
	Clindamycin or	600 mg *t.i.d*. oral	10–11 weeks
	Minocyclin or	100 mg *b.i.d*. (first dose 200 mg) oral	10–11 weeks
	Co-trimoxazole	960 mg *t.i.d*. oral	10–11 weeks
	All in combination with Rifampin^b^	450 mg *b.i.d*. oral	10–11 weeks
	In the case of linezolid, use only as monotherapy (not combined with rifampin)	600 mg* b.i.d*. oral	max. 6 weeks, then switch to alternative
***Streptococci***	Benzylpenicillin	2 million units loading dose, followed by 12 million units/24 h continuous infusion IV	2 weeks
	or		
	Ceftriaxone	2.000 mg once daily IV *or* 2.000 mg loading dose, followed by 2.000 mg/24 h continuous infusion IV	2 weeks
	± Rifampin [20]^b^	450 mg *b.i.d*. oral	12 weeks
	followed by:		
	Amoxicillin	750–1000 *t.i.d*. mg oral	10 weeks
	± Rifampin [20]^b^	450 mg *b.i.d*. oral	10 weeks
***Enterococci*** **(amoxicillin-sensitive)**	Amoxicillin	2.000 mg loading dose, followed by 12.000 mg/24 h continuous infusion IV	4 weeks
	plus		
	Ceftriaxone	2.000 mg *b.i.d*. IV *or* 2.000 mg loading dose, followed by 4.000 mg/24 h continuous infusion	4 weeks
	followed by:		
	Amoxicillin	750–1000 mg *t.i.d*. oral	8 weeks
***Enterococci*** **(amoxicillin-resistant)**	Vancomycin	20 mg/kg loading dose, followed by 30 mg/kg/24 h continuous infusion (with adjustments based on blood level monitoring)	6 weeks
	plus		
	Gentamicin^#^	3 mg/kg *q.d*. IV (with adjustments based on blood level monitoring)	2 weeks
	followed by:		
	Linezolid^§^	600 mg *b.i.d*. oral (preferably with blood level monitoring)	6 weeks
***Enterobacteriaceae (e.g. E. coli, Klebsiella, Proteus)***	Ceftriaxone	2.000 mg once daily IV *or* 2.000 mg loading dose, followed by 2.000 mg/24 h continuous infusion	1–2 weeks
	followed by:		
	Ciprofloxacin	750 mg *b.i.d*. oral	10–11 weeks
	In case of ciprofloxacin resistance:		
	Cotrimoxazole	960 mg* t.i.d*. oral	10–11 weeks
**Nonfermenters** (***e.g. Pseudomonas aeruginosa)***	Ceftazidime	2.000 mg *t.i.d*. IV *or* 2.000 mg loading dose, followed by 6.000 mg/24 h continuous infusion IV	1–2 weeks
	plus		
	Ciprofloxacin	400 mg *t.i.d*. IV (or directly 750 mg b.i.d. oral)	1–2 weeks
	followed by:		
	Ciprofloxacin	750 mg *b.i.d*. oral	10–11 weeks
***Cutibacterium acnes (Propionibacterium acnes)***	Benzylpenicillin	2 million units loading dose, followed by 12 million units/24 h continuous infusion IV	1–2 weeks
	or		
	Ceftriaxone	2.000 mg once daily IV *or* 2.000 mg loading dose, followed by 2.000 mg/24 h continuous infusion	1–2 weeks
	followed by:		
	Amoxicillin	750–1000 mg* t.i.d*. oral	10–11 weeks
	or		
	Clindamycin	600 mg *t.i.d*. oral	10–11 weeks
***Corynebacterium species***	Vancomycin	20 mg/kg loading dose, followed by 30 mg/kg/24 h continuous infusion (with adjustments based on blood level monitoring)	1–2 weeks
	followed by:		
	Cotrimoxazole	960 mg *t.i.d*. oral	10–11 weeks
	or		
	Clindamycin	600 mg* t.i.d*. oral	10–11 weeks
	or		
	Minocyclin	200 mg loading dose, followed by 100 mg b.i.d oral	10–11 weeks
	If resistant for the above:		
	Vancomycin	20 mg/kg loading dose, followed by 30 mg/kg/24 h continuous infusion (with adjustments based on blood level monitoring)	6 weeks
	or		
	Dalbavancin	1500 mg once every two weeks	6 weeks (3x)
	followed by:		
	Linezolid^d^	600 mg *b.i.d*. oral	6 weeks
**Candidal species (fluconazol-sensitive)**	Caspofungin	70 mg IV loading dose, followed by 50 mg *q.d*. if < 80 kg, and 70 mg *q.d*. if > 80 mg	2–4 weeks^e^
	followed by:		
	Fluconazole	800 mg loading dose, followed b 400 mg *q.d*. oral	5 months
	In the case of 2-stage revision:		
	500 mg conventional amfotericin B or 200 mg liposomal amfotericin B in cement spacer^f^		

^a^The above doses are based on adequate kidney function and a normal weight/BMI. In the case of deviating values, contact the hospital pharmacist for dosing advice

^b^Only start with a dry wound and proven susceptibility. The addition of rifampin in a streptococcal infection is advised in a DAIR where the mobile components cannot be replaced.

^c^Total treatment duration 3 months (except for Candida)

^d^Do not prescribe longer than 4 weeks, unless therapeutic drug monitoring is performed.

^e^4 weeks in the case of a DAIR, 2 weeks in the case of extraction of the prosthesis

^f^This dosage should only be used in the temporary cement spacer and not in the fixation cement (There are not sufficient clinical data to guarantee the stability of the cement.)

#### Treatment duration

As a rule, high-dose intravenous antibiotics are started for the treatment of planktonic bacteria. In general, this is administered for a period of 7–14 days. If the patient is showing a good clinical recovery with a rapidly decreasing CRP and if an oral antibiotic with good bioavailability can be administered, 7 days of IV therapy is considered sufficient. *Enterococci* and *Candida* infections always require a total IV treatment duration of 4 weeks, and in the case of *Streptococci* infection, the duration should be 2 weeks, before switching to an oral regimen, since the bioavailability and/or antibiofilm effect of the available oral agents are limited.

The oral antibiotic regimen targets the bacteria that are in the stationary phase (in a biofilm), and requires a total duration of 12 weeks (including IV therapy). Some studies have shown good outcomes using a treatment duration of only 6 weeks. However, most of them have been performed in a selected group of patients [18]. In addition, a 6-week treatment showed a worse outcome in a recent randomized trial from France [19], especially in acute infections treated with DAIR.

#### Antibiotic course during a two-stage revision

**Fig. 3:**
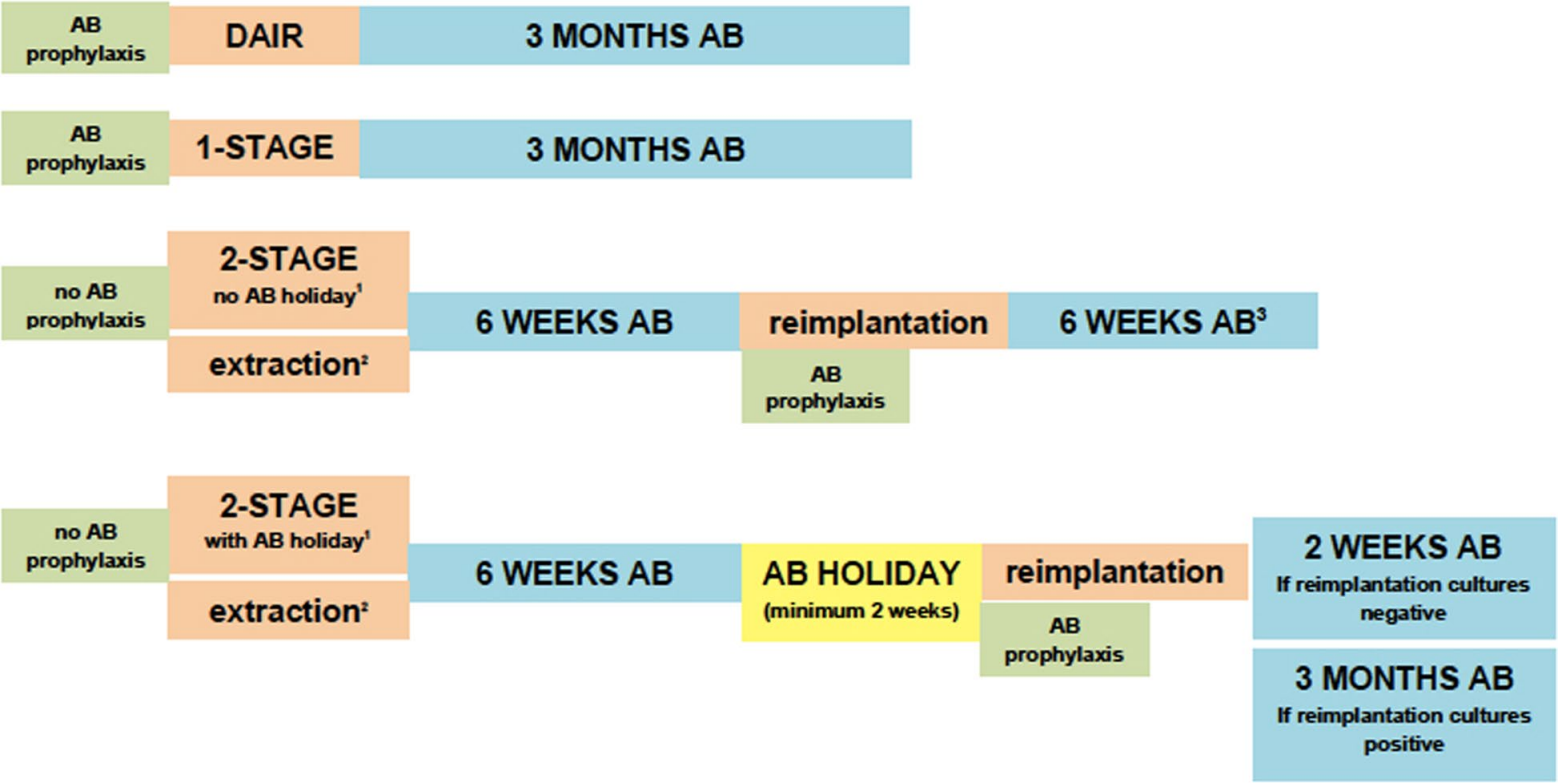
Surgical strategy in relation to antibiotic treatment duration. ^1^A 2-stage exchange without antibiotic holiday is preferred. ^2^Targeted antibiotics in spacer (incl. vancomycin regardless of causative agent). If the causative agent is unknown, the most commonly used regimen is: gentamicin and vancomycin. Prefabricated spacers often contain gentamicin and clindamycin in the cement, but previous studies have shown that adding vancomycin (2 g per 40 g of cement) reduces secondary spacer infections with coagulase-negative *Staphylococci* [10], and is therefore recommended to be added. In general, it should be taken into account that the stability of the cement is adversely affected if more than 10% antibiotics are added to the cement (= 4 g of antibiotics per 40 g of cement). This is especially important with cement fixation (and less so when using temporary cement spacers). ^3^Provided that reimplantation cultures are negative

In the case of a 2-stage revision, the prosthesis is, in general, re-implanted after 6 weeks of antibiotic treatment (under oral antibiotic treatment and standard antibiotic prophylaxis), followed by an additional 6 weeks of antibiotic treatment aimed at the initial causative microorganism (Fig. 3). In the case of positive intraoperative cultures with rifampin-sensitive staphylococcal species at the time of reimplantation, we advise to additionally start rifampin as a co-drug for a total duration of 3 months. We do not advise rifampin in a Girdlestone situation, nor when cement spacers are present. The antibiotic policy regarding reimplantation must be coordinated within the multidisciplinary team.

While on antibiotic treatment, it is important to monitor the patient for possible side-effects (*e*.*g*., bone marrow depression, kidney failure) by performing scheduled outpatient follow-up visits with biochemical monitoring.

## Conclusions

Periprosthetic joint infections are increasingly common and their treatment remains challenging and requires interdisciplinary collaboration between infectious disease specialists, experienced orthopedic surgeons and medical microbiologists. The establishment of our NINJA diagnostic and treatment protocol for PJI in our region has enabled mutual understanding, has supported agreement on how to treat specific patients, and has led to clarity for smaller hospitals in our region for when to refer patients, without jeopardizing important initial treatment locally. Furthermore, the establishment of a mutual PJI patient database has enabled improvement of our protocol, based on medicine-based evidence from own scientific data (*e*.*g*., the use of calprotectin in the diagnostic work-up, abandoning the use of gentamicin beads and/or fleeces during DAIR, determining the maximum interval between the index surgery and DAIR, and the indications for a second DAIR [6,16, 17, 27]). Although the continuous registration of data is labor-intensive, it is of vital importance for innovation and progress in the care of vulnerable and complex PJI patients.

## Data Availability

Not applicable.

## Abbreviations

PJI: Periprosthetic joint infection
NINJA: Northern Infection Network for Joint Arthroplasty
DAIR: Debridement Antibiotics and Implant Retention
CRP: C-reactive protein
S. Aureus: *Staphylococcus Aureus*
MSIS: Muskuloskeletal Infection Society
ESR: Erythrocyte Sedimentation Rate
ICM: International Consensus Meeting
KLIC: Kidney, Liver cirrhosis, Index surgery, C-reactive, protein, Cemented prosthesis
CRIME80: Chronic obstructive pulmonary disease, CRP, rheumatoid arthritis, index surgery, exchange of mobile components, age > 80
IV: Intravenous
